# 2903. Early Experience With Omadacycline For The Treatment Of Diabetic Foot Infections

**DOI:** 10.1093/ofid/ofad500.174

**Published:** 2023-11-27

**Authors:** Matthew P Crotty, Ronda L Akins, Edward A Dominguez, Julie Alexander, Nebu Alexander

**Affiliations:** Methodist Dallas Medical Center, Dallas, Texas; Methodist Charlton Medical Center, Dallas, Texas; Methodist Transplant Physicians, Dallas, TX; Methodist Dallas Medical Center, Dallas, Texas; Methodist Charlton Medical Center, Dallas, Texas

## Abstract

**Background:**

Diabetic foot infections (DFI) are often polymicrobial and increasingly caused by resistant pathogens. Progression of resistance requires utilization of broad-spectrum agents, further exacerbating the problem and resulting in collateral damage. Frequently used regimens are often complicated by adverse effects of acute kidney injury (AKI) and *Clostridioides difficile* infection (CDI). The unique spectrum of activity of omadacycline (OMC), availability both intravenously (IV) and orally, along with its safety profile may provide an optimal treatment for DFIs.

**Methods:**

This is a prospective, open-label, two-center study (NCT04714411) to assess the safety of OMC use in the treatment of hospitalized subjects with moderate to severe DFI with or without acute osteomyelitis (AOM, defined as initial diagnosis) who are at high risk for development of CDI, AKI, and/or resistant pathogens. Adults (≥18 years) hospitalized with DFI were eligible. All subjects received OMC 200 mg IV once on day 1 followed by either 100 mg IV or 300 mg by mouth once daily for 14-21 days without AOM or 42 days with AOM. Clinical success was defined as infection resolution or sufficient clinical improvement requiring no other antibiotics.

**Results:**

11 patients were enrolled and received OMC for DFI treatment (Table 1). Most patients had AOM (8/11, 72.7%) and systemic inflammatory response syndrome criteria were met in 5 of 11 (45.5%). All patients were converted to oral therapy with median time of 4 days (IQR, 2-5 days). Median hospital stay was 11 days (IQR, 9-15). 8 patients completed treatment with all achieving clinical success at end of therapy (EOT; Figure 1). At test-of-cure (TOC; EOT + 30 days), 6/8 (75%) patients had clinical success. Among two patients not achieving clinical success, one received additional antibiotic therapy during a subsequent hospitalization and the other had growth of *Proteus mirabilis* from local culture. At long-term follow-up (LFU; EOT + 60 days), 6/8 (75%) achieved clinical success. No instances of AKI or CDI were observed. Adverse effects were reported by two patients (nausea and burning sensation at injection site).

**Figure:**
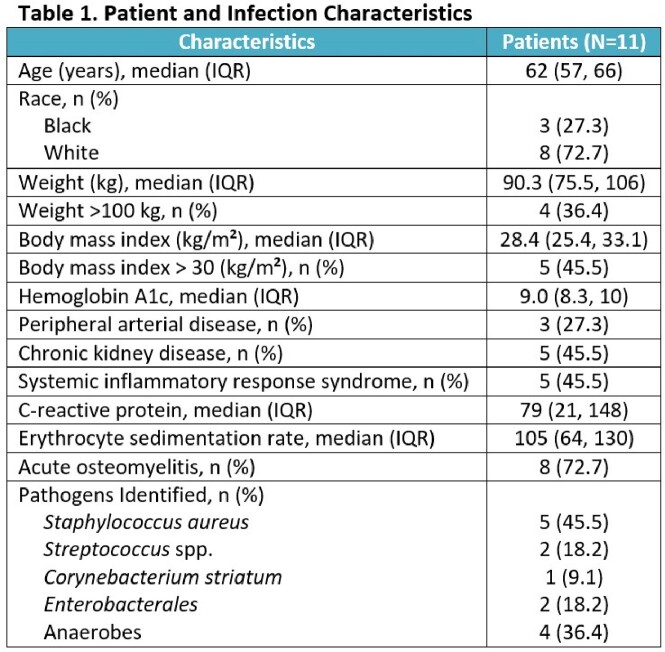

**Figure:**
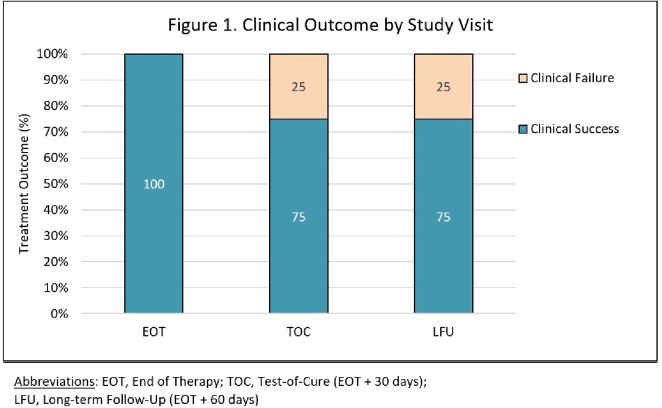

**Conclusion:**

Early experience with OMC suggests it may be a potential treatment for some patient with DFI including AOM. Continued investigation for this indication is warranted.

**Disclosures:**

**Matthew P. Crotty, PharmD, BCIDP**, Paratek Pharmaceuticals Inc.: Grant/Research Support **Ronda L. Akins, PharmD**, Paratek Pharmaceuticals, Inc.: Grant/Research Support

